# Cryo-Electron Microscopy Structure of the *Macrobrachium rosenbergii* Nodavirus Capsid at 7 Angstroms Resolution

**DOI:** 10.1038/s41598-017-02292-0

**Published:** 2017-05-18

**Authors:** Kok Lian Ho, Chare Li Kueh, Poay Ling Beh, Wen Siang Tan, David Bhella

**Affiliations:** 1 Department of Pathology, Faculty of Medicine and Health Sciences, Universiti Putra Malaysia, 43400 UPM Serdang, Selangor Malaysia; 2 Department of Microbiology, Faculty of Biotechnology and Biomolecular Sciences, 43400 UPM Serdang, Selangor Malaysia; 3 MRC-University of Glasgow Centre for Virus Research, Sir Michael Stoker Building, Garscube Campus, 464 Bearsden Road, Glasgow, G61 1QH Scotland UK

## Abstract

White tail disease in the giant freshwater prawn *Macrobrachium rosenbergii* causes significant economic losses in shrimp farms and hatcheries and poses a threat to food-security in many developing countries. Outbreaks of *Macrobrachium rosenbergii* nodavirus (*Mr*NV), the causative agent of white tail disease (WTD) are associated with up to 100% mortality rates. There are no interventions available to treat or prevent *Mr*NV disease however. Here we show the structure of *Mr*NV virus-like particles (VLPs) produced by recombinant expression of the capsid protein, using cryogenic electron microscopy. Our data show that *Mr*NV VLPs package nucleic acids in a manner reminiscent of other known nodavirus structures. The structure of the capsid however shows striking differences from insect and fish infecting nodaviruses, which have been shown to assemble trimer-clustered T = 3 icosahedral virus particles. *Mr*NV particles have pronounced *dimeric* blade-shaped spikes extending up to 6 nm from the outer surface of the capsid shell. Our structural analysis supports the assertion that *Mr*NV may belong to a new genus of the *Nodaviridae*. Moreover, our study provides the first structural view of an important pathogen affecting aquaculture industries across the world.

## Introduction

*Macrobrachium rosenbergii* nodavirus (*Mr*NV) causes white tail disease (WTD) in the giant freshwater prawn *M. rosenbergii. Mr*NV outbreaks in shrimp farms and hatcheries can result in mortality rates of up to 100%, thus the virus represents a threat to food-security and causes significant economic losses^1^. WTD disease was first reported in Pointe Noire, Guadeloupe in 1997, and was subsequently detected in China^2^, India^3^ Taiwan^4^, Thailand^5^, Malaysia^6^, Australia^7^ and most recently in Indonesia^8^.

*Mr*NV belongs to the family *Nodaviridae*, which is divided into *Alphanodavirus* and *Betanodavirus* genera. The former includes insect-infecting nodaviruses such as Nodamura virus (NoV), Boolaara virus (BoV), Flock House virus (FHV), black beetle virus (BBV) and Pariacoto virus (PaV). The latter comprises fish-infecting nodaviruses such as Malabaricus grouper nervous necrosis virus (MGNNV) [see Ref. 6 for a review]. Based on the amino acid sequence of the RNA dependent RNA polymerase (RdRp), *Mr*NV is grouped within the *Alphanodavirus* genus but owing to some unique characteristics of *Mr*NV, a new genus (*Gammanodavirus*) has been proposed which would comprise *Mr*NV and *Penaeus vannamei* nodavirus (*Pv*NV)^9^. In common with other nodaviruses, *Mr*NV is a non-enveloped, icosahedral virus which comprises a nucleocapsid containing a bipartite positive-sense RNA genome. The genomic RNAs: RNA 1 (3.2 kb) and RNA 2 (1.2 kb), encode the RdRp and capsid protein, respectively. The full length *Mr*NV capsid protein is a polypeptide containing 371 amino acids and having an N-terminal region rich in positively charged amino acids that is believed to be an RNA binding region^10^. The C-terminal region of the capsid protein is involved in host cell binding and internalization^11^. This feature allows foreign epitopes such as the ‘*a*’ determinant of hepatitis B surface antigen (HBsAg) and the ectodomain of influenza A virus M2 to be inserted at the C-terminal region and thereby displayed on the surface of the *Mr*NV capsid^12, 13^. Other functional regions within the polypeptide of *Mr*NV capsid protein have not been clearly identified.

Recombinant *Mr*NV capsid protein produced in *Escherichia coli* and *Spodoptera frugiperda* (both *Sf*9 and *Sf*21) assembles into icosahedral virus-like particles (VLPs) although these were found to be morphologically different under transmission electron microscopy analysis^14, 15^. Negative stained VLPs expressed in *E. coli* and *Sf*9 were measured to have diameters of ~30 and 40 nm, respectively^14, 15^. The self-assembly/disassembly capability of the *Mr*NV capsid protein has been exploited to develop nano-carriers for DNA and double stranded RNA^16, 17^. Owing to the economic importance of *Mr*NV and the potential applications of recombinant capsid proteins, there is considerable interest in the three-dimensional (3D) structure of the *Mr*NV nucleocapsid. Determining the structure will provide insights into the molecular details of virion morphogenesis, potentially leading to the identification of *Mr*NV inhibitors suitable for use in combating *Mr*NV infection. To date, the high-resolution structures of several alphanodaviruses including FHV^18^, PaV^19^, BBV^20^ and NoV^21^ have been solved by X-ray crystallography. Of the betanodaviruses, a high-resolution structure has been determined for grouper nervous necrosis virus (GNNV)^22^. A structure for the unclassified Orsay virus, which infects the nematode worm *Caenorhabditis elegans*, has also been determined^23^. All known structures of *Nodaviridae* reveal T = 3 icosahedral capsids assembled from 180 capsid proteins that have an eight-stranded antiparallel beta-barrel topology. Known nodavirus capsid structures display trimer-clustering; PaV and NoV having small trimeric spikes on their outer surface, while Orsay and GNNV have larger trimeric capsomeres.

Amino acid sequence alignment showed that FHV capsid protein shares ~87% sequence identity with BBV capsid protein. However, none of these nodavirus capsid proteins show similarities beyond 20% with *Mr*NV or *Pv*NV capsid proteins. Here, we report a 3D reconstruction of *Mr*NV VLPs determined by cryo-electron microscopy at a resolution of 7 Å. The 3D reconstruction reveals a T = 3 icosahedral assembly that shows a striking divergence from the known structures of other nodavirus capsids. This was characterized by the presence of large dimeric blade-like spikes on the outer surface. Although the VLPs assembled in a heterologous system and in the absence of the full-length viral genome, we find that the capsids contain density consistent with the encapsidation of RNA, which we assume to be the cognate mRNA. Overall, the considerable morphological differences seen when comparing the structure of *Mr*NV to other known nodaviruses supports the assertion that this virus may belong to a new genus of nodaviruses.

## Results

### *Mr*NV VLPs assemble as dimer-clustered T=3 icosahedral capsids that package nucleic acids

**Figure 1:**
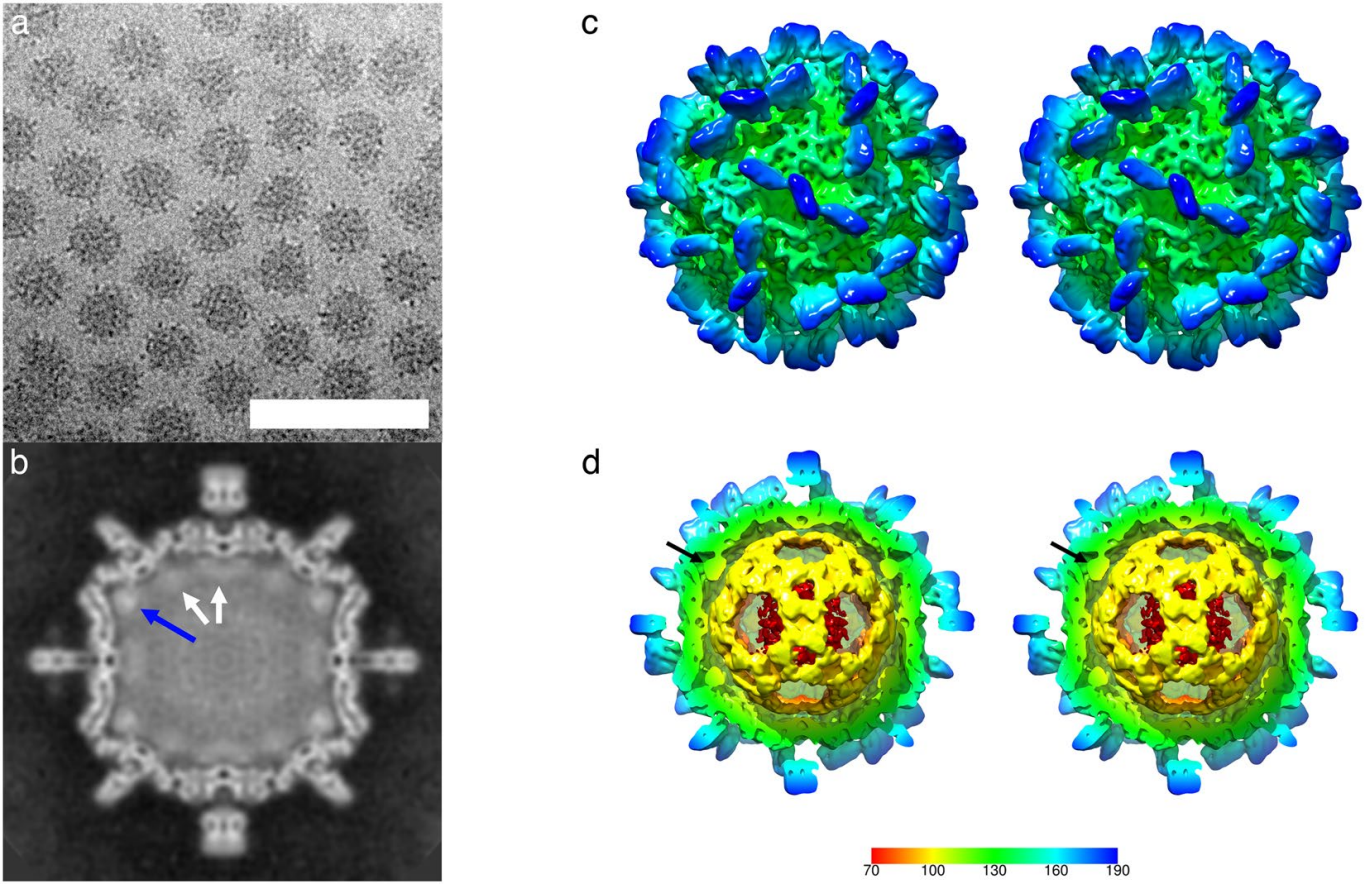
Cryo-Electron microscopy of *Mr*NV virus-like particles reveals a population of spikey icosahedral particles that package nucleic acids. (**a**) Cryo-EM of *Mr*NV VLPs revealed a population of isometric particles with pronounced spikes on the outer surface. VLPs were dense in projection, indicating that they packaged nucleic acids (Scale bar 100 nm). (**b**) A central section through the reconstructed *Mr*NV VLP density map shows a clearly defined capsid structure with pronounced spikes on the outer surface. Lining the capsid interior is a region of fuzzy density (white arrows) that we attribute to packaged RNA. The strongest density is seen to lay under the icosahedral five-fold symmetry axes and may represent RNA or a poorly-ordered N-terminal region of the capsid protein (blue arrow). (**c**) A wall-eyed stereo-pair image of the isosurfaced 3D reconstruction highlights the overall particle morphology featuring pronounced dimeric spikes on the capsid surface at the two-fold symmetry axes and arranged about the five-fold axes. (**d**) A cut-away view reveals that the internal density highlighted in (**b**) forms a dodecahedral cage, a feature previously documented in other nodavirus structures. The density underlying the five-fold symmetry axes is not, however, contiguous with this structure (black arrow). The colour key indicates the scale (in angstroms) of the radial colour scheme used in Figs 1(c,d), 2 and 3(a,c).

To investigate the structure of the *Mr*NV capsid, VLPs were produced by expression of the capsid gene in a recombinant baculovirus system and purified from *Sf*9 cells by differential ultracentrifugation. VLPs were prepared for cryo-electron microscopy by plunge freezing in liquid-ethane. Figure 1(a) shows a representative cryomicrograph revealing a population of particles with pronounced spikes on their outer surface and that measured 40 nm in diameter. VLPs appeared dense in projection, suggesting that they contain nucleic acids. Two hundred and eighty-one cryo-micrographs of *Mr*NV VLPs were recorded for 3D image reconstruction. Automated particle picking was used to extract a dataset of 9592 particles. These data were processed to calculate a reconstruction using the RELION software package. Following 2D and 3D classification procedures, 1658 particles were retained for the final round of refinement, achieving a resolution of 7 angstroms (Supplemental Fig. 1).

Figure 1(b) shows a central slice through the reconstruction prior to sharpening and Fig. 1(c) is a stereo-pair view of the isosurfaced reconstruction, also unsharpened. The density map reveals a particle with pronounced, blade-like, 6 nm spikes on the outer surface that are located at the icosahedral two-fold symmetry axes and about the five-fold axes. The location of these spikes is consistent with dimer-clustered T = 3 icosahedral symmetry. Thus, we shall adopt the domain nomenclature used when describing similar structures, such as capsids of the *Caliciviridae*. We divide the capsid protein into protruding (P) domain and shell (S) domain^24^. Likewise, we shall describe the capsid structure as having dimers assembled from three quasi-equivalent capsid proteins termed A, B and C. These are arranged as AB dimers (about the five-fold axes) and CC dimers (located at the two-fold axes).

Inspection of Fig. 1(b) reveals a clearly defined capsid shell measuring between 2 and 3.5 nm thick. Lining the capsid interior is a less well-resolved fuzzy density (white arrows) that is particularly bright beneath the five-fold symmetry axes (blue arrow). We assume this to be packaged nucleic acids, most likely RNA, the density beneath the five-fold axes could be RNA or less well-ordered regions of the capsid protein. In Fig. 1(d) the reconstruction is cropped to reveal the internal density, showing the presence of a dodecahedral cage structure. Similar features have been described previously in cryo-EM studies of nodaviruses and other positive sense RNA containing viruses^19, 25–28^. The density below the five-fold symmetry axes is not, however, contiguous with this feature (black arrow).

### *Mr*NV P-domains form blade-like dimers

The most striking feature of the *Mr*NV capsid is the presence of large blade-like P-domains. These features are well resolved in the reconstructed density, and show marked differences between spikes at the two-fold axes (CC dimers) and those about the five-fold axes (AB dimers).

**Figure 2:**
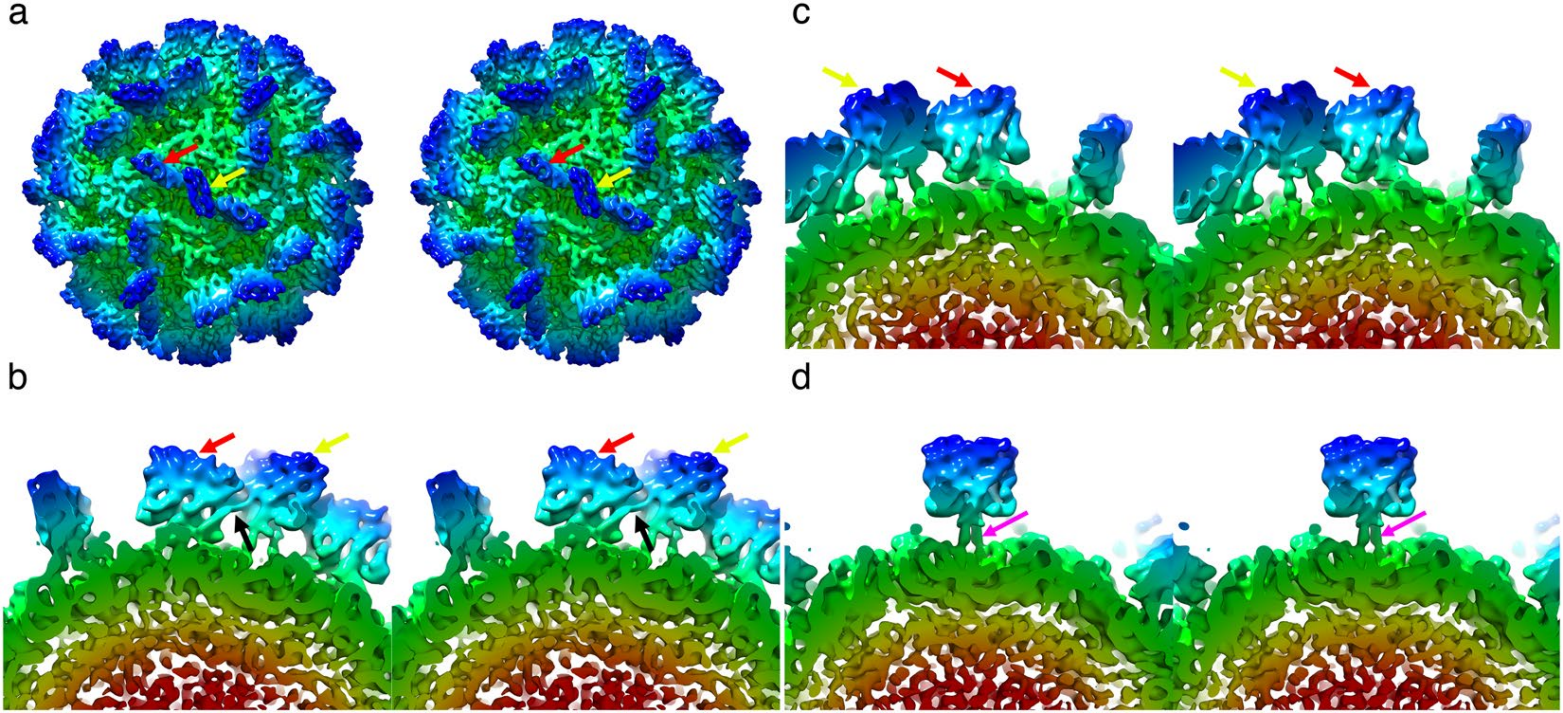
*Mr*NV particles have pronounced blade-shaped dimeric protrusions. (**a**) Stereo view of the sharpened 3D reconstruction of the *Mr*NV VLP showing the location of AB (red arrow) and CC (yellow arrow) P-dimers. (**b**) An AB P-dimer (red arrow), viewed parallel to the capsid surface, showing that the protruding spike leans towards the icosahedral two-fold symmetry axis, making contact with the CC-dimer, which is identified by a yellow arrow, the point of contact is highlighted with a black arrow. (**c**) A view of the same AB P-dimer shown in (**b**), but rotated by 180°, highlighting the two-fold symmetry of this feature – a property that is not imposed by the 3D reconstruction process. (**d**) unlike the AB-dimer, the CC dimer is oriented perpendicular to the capsid surface and raised on two clearly resolved legs of density (pink arrow).

Figure 2(a) shows the *Mr*NV VLP reconstruction following a sharpening procedure that reduces the influence of low-resolution information on the isosurface representation, thus enhancing high-resolution detail. A close-up view of the AB and CC dimers is presented in Fig. 2(b,c) and (d) respectively. These images show that the P-domain dimers form square-shaped blades that are 4.7 nm in both width and height. The spikes are however very thin, measuring only 1.8 nm in depth. These structures are clearly two-fold symmetric as can be seen in Fig. 2(b) and (c), in which the AB spike is rotated 180° (unlike the CC spike, rotational symmetry is not applied to this feature by the icosahedral reconstruction process as it is located at a quasi-two-fold symmetry axis). The density of the spikes shows comparable features in both AB and CC dimers except that the P-domains are quite differently oriented relative to their respective S-domains. Firstly, the CC P-dimers are raised from the capsid surface on two well resolved legs of density (pink arrow) and with a clearance of approximately 2 nm from the outer surface of the capsid shell. The AB P-dimers sit much closer to the outer surface of the capsid shell and are tilted approximately 20° towards the icosahedral two-fold symmetry axes such that on the two-fold proximal side of the feature, there is ~0.5 nm between the P-domains and the S-domains, while on the 2-fold distal side, a gap of ~1.5 nm separates them. At this resolution, the AB P-dimers appear to contact their nearest CC P-dimers (black arrow Fig. 2b), creating a larger structure comprised of three P-dimers (two AB P-dimers and one CC P-dimer).

**Figure 3:**
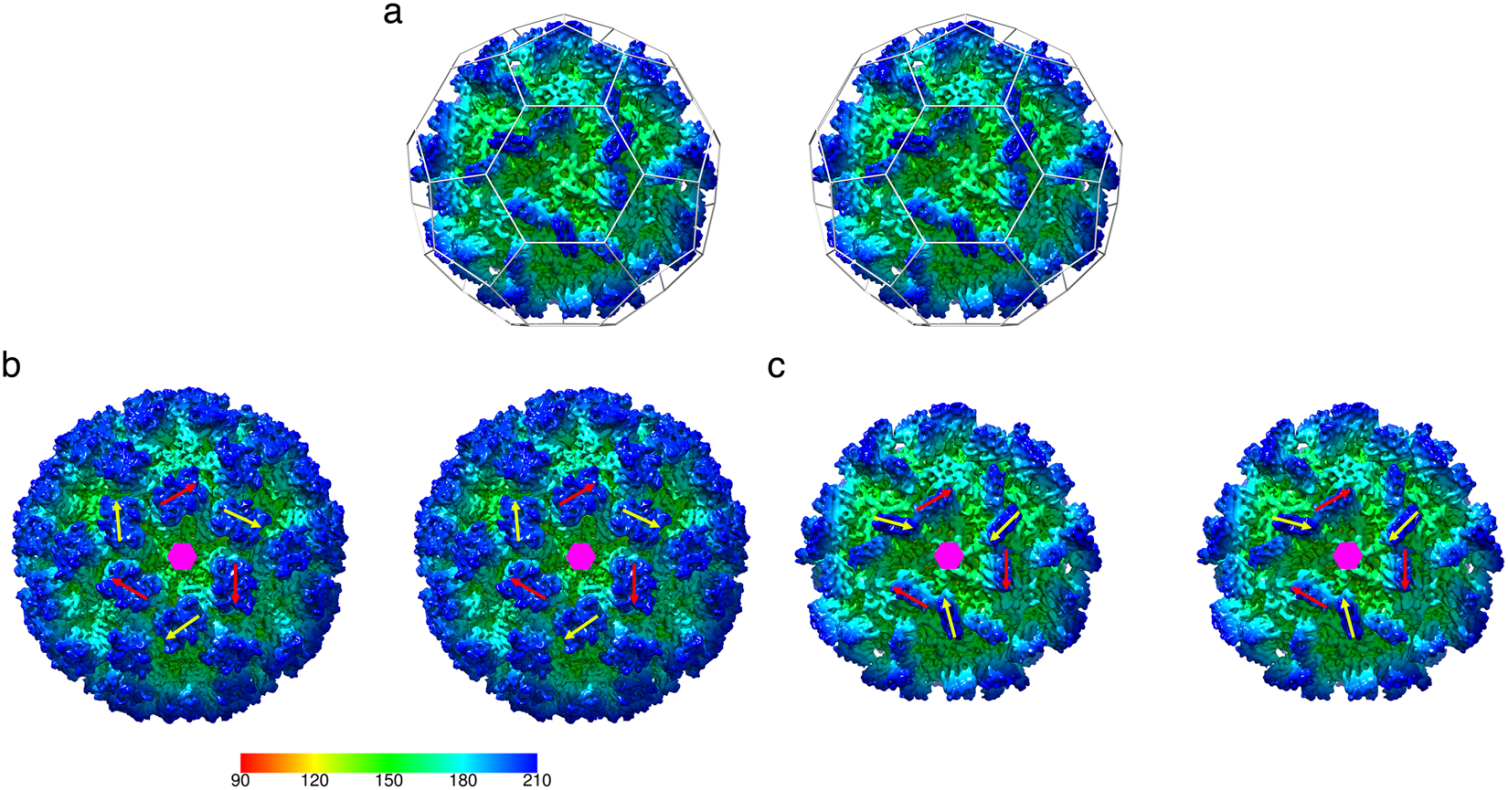
*Mr*NV P-domains show a pronounced rotation between AB and CC dimers – the quasi-equivalent positions for dimeric capsomeres in a T = 3 icosahedral virus capsid. (**a**) A stereo pair of the *Mr*NV reconstruction viewed along an icosahedral three-fold symmetry axis. In T = 3 icosahedral capsids, a local six-fold quasi-symmetry axis lies at this point. The locations of icosahedral five-fold and local quasi-six-fold symmetry axes are highlighted by a ‘soccer-ball’ cage. (**b**) Dimer clustered T = 3 icosahedral capsids, such as our recently published structure for feline calicivirus have AB and CC P-dimers arranged alternating about the quasi-six-fold axes. Here the positions of such are indicated by red arrows (AB) and yellow arrows (CC). If the structures of AB and CC dimers are not radically different, we expect the position and orientation of these features to be related by an approximately 60° rotation about the local six-fold symmetry axis (indicated by a pink hexagon). (**c**) In the case of the *Mr*NV P-dimers, the CC P-dimers are rotated approximately 105° relative to the orientation of the AB P-dimers. The radial colour scheme in panel (**b**) indicates the radial depth cue scale in angstroms for the FCV reconstruction only.

Another striking feature of the P-domains is that there is a pronounced rotation in the orientations of the AB and CC P-dimers relative to the underlying S-domains and the icosahedral symmetry axes. Figure 3(a) shows a stereo-pair image of the *Mr*NV VLP viewed along an icosahedral three-fold symmetry axis. In T = 3 icosadeltahedra, quasi-six-fold symmetry axes are located at the icosahedral three-fold axes, as highlighted by the ‘soccer ball’ cage of pentagons and hexagons indicating the locations of icosahedral five-fold and local six-fold symmetry axes. We therefore expect gross morphological features, such as domain orientation to follow this local six-fold symmetry as is seen in the dimer-clustered T = 3 capsids encoded by the *Caliciviridae*. Figure 3(b) shows a structure for feline calicivirus (FCV) that we have recently determined^29^. In this reconstruction, the orientation of the CC P-dimer (yellow arrow) is approximately related to that of the AB P-dimer (red arrow) by a 60° rotation about the local six-fold symmetry axis (indicated by a pink hexagon). Figure 3(c) highlights the relative orientations of the P-domains for *Mr*NV, showing that the orientations of the CC P-dimers deviate by approximately 105° relative to that of the AB P-dimers, such that the yellow arrows indicating the orientations of the CC P-dimers point towards the local six-fold axis, while the red arrows, marking the orientations of the AB P-dimers, point clockwise relative to this axis.

## Discussion

*Macrobrachium rosenbergii* nodavirus is an important pathogen that poses both an economic and a food-security threat to the growing fresh-water aquaculture industries of many developing countries. We sought to generate structure data that would support the development of interventions to treat or prevent outbreaks of *Mr*NV disease. Such incidents are associated with mortality rates of up to 100% owing to the ease with which waterborne pathogens can spread among the dense populations found in shrimp farms.

We used cryogenic transmission electron microscopy to image purified *Mr*NV VLPs in a frozen hydrated state. Processing the resulting images led to the calculation of a 7 Å resolution 3D reconstruction of the capsid.

We found that expression of the recombinant capsid protein in insect cells led to the production of VLPs that when reconstructed showed density reminiscent of packaged viral genomes in known nodavirus structures^19, 25^. We hypothesize that this density is packaged nucleic acid, possibly the cognate mRNA. During authentic infections, positive-sense RNA containing viruses do not express their genetic content via the production of mRNA, rather their genomes are directly translated by host ribosomes to produce the encoded gene-products. There is growing evidence that capsid assembly in many positive-sense RNA viruses is directed by specific encapsidation signals throughout the viral genomic RNA^30^. It is possible then that such encapsidation signals may also be present in mRNA produced in experiments such as ours, in which a DNA sequence was generated from the viral genome for recombinant expression.

Although our reconstruction is insufficient to determine the tertiary structure of the capsid protein, it has revealed a striking divergence from known nodavirus structures. Most notably, while other known nodavirus virions exhibit *trimer*-clustering of capsid proteins arranged with T = 3 icosahedral symmetry^19, 22, 25, 26^, our map shows pronounced *dimeric* capsomeres. Our data further revealed a capsomere morphology that is very different from known similar capsids. Firstly, the spikes we observed were seen to form as approximately square, thin blade-shaped protrusions. Moreover, there was considerable difference in the orientation of these capsomeres relative to each other. The AB P-domains were seen to lean towards the icosahedral two-fold symmetry axes, while the CC P-domains were raised away from the capsid shell. Furthermore, the lean resulted in the AB P-dimers contacting the CC P-dimers to form a larger blade-like super-structure lying across the icosahedral two-fold axes. There was also a rotation of 105° in the quasi-symmetry related orientations of AB and CC P-domains, possibly a consequence of the contact between the two dimer forms.

**Figure 4:**
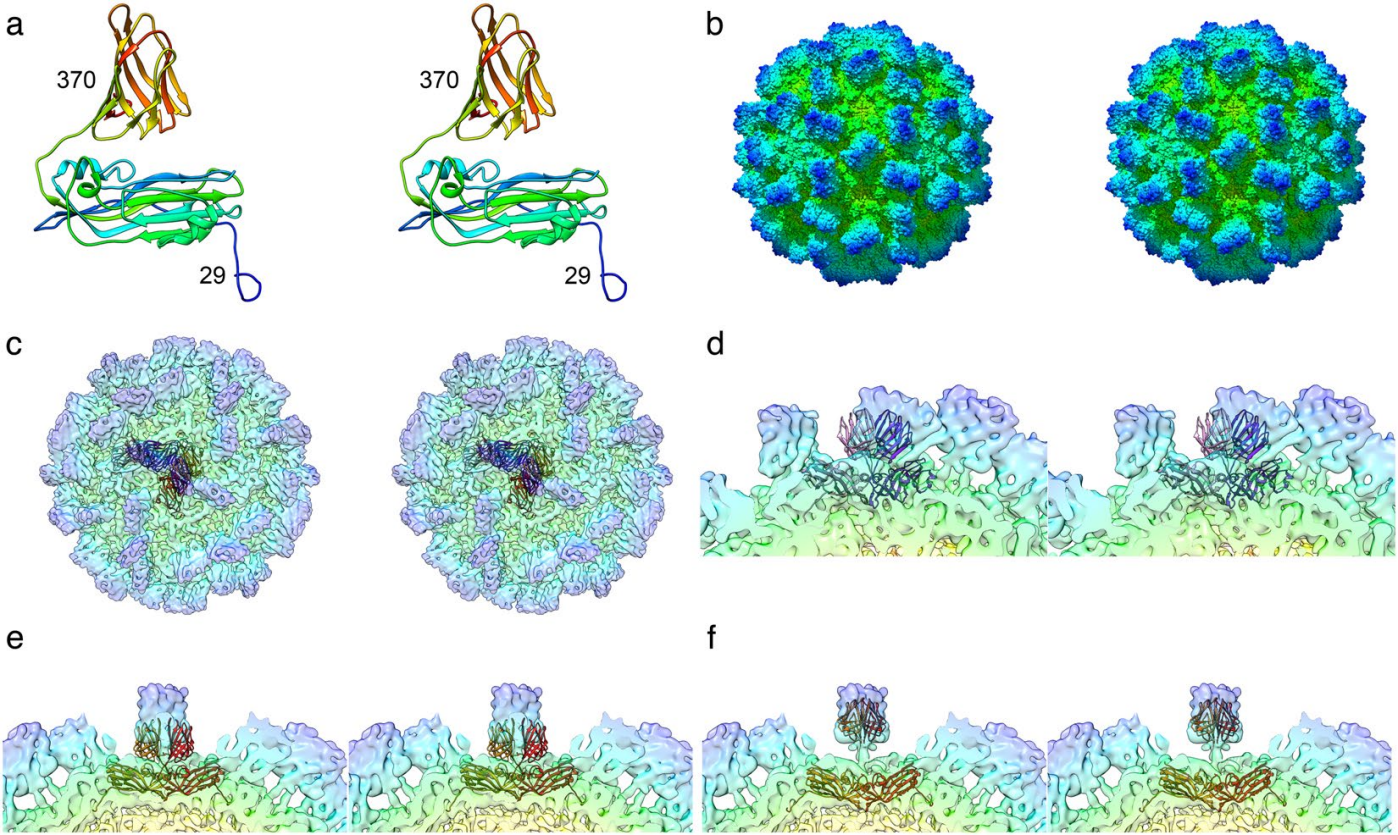
Homology modelling of the *Mr*NV capsid protein. Structure prediction has previously been used to compute a model for the MrNV capsid protein by homology modelling against cucumber necrosis virus (CNV) – PDB ID 4LLF. To test whether this model was compatible with our reconstructed density map, we repeated the modelling. Stereo pair views are presented. (**a**) A ribbon diagram of the homology model shows the reported two-domain structure. The N and C terminal residues are labelled indicating the extent of the capsid sequence that was modelled. (**b**) The solvent excluding surface representation of the CNV capsid structure that informed the homology model reveals a clear dimer-clustered T = 3 assembly. (**c**) A transparent view of the MrNV reconstruction, into which we have inserted AB dimers (purple/pink) and CC dimers (red/orange) of the homology model. A close-up view of the AB-dimer (**d**) shows that there is poor correlation between the positions of the P domain in the homology model and reconstructed density map. Likewise, the reconstructed density for the MrNV P-domain at the CC position is also differently positioned from that predicted by the homology model, the feature is significantly raised from the capsid surface and rotated (**e**). Rigid-body fitting of the individual S and P domains at the CC-dimer position gave reasonable results, although the shape of the CNV P-domain is broader than the thin, blade-like structure in MrNV (**f**).

It has previously been reported that the C-terminal domain of the *Mr*NV capsid protein likely encodes the functional attachment and entry requirements of the virus^11^, while the N-terminal region contains positive charged residues that bind the viral genome^10^. Our structure shows that the CC P-dimer is supported on two narrow legs of density. Taken together these data strongly suggest that the P-domain is not an insert in the capsid polypeptide chain rather it is at the C-terminus. The presence of a discrete dimeric P-domain comprising the C-terminal region of the capsid protein was proposed by Somrit *et al*.^11^. In that study web-based homology modelling software was used to predict the structure of the *Mr*NV capsid protein. Protein sequence similarity analysis identified the capsid protein of a tombusvirus - cucumber necrosis virus as a suitable template. A model based on the known crystal structure of that protein^31^ was calculated, producing a predicted two-domain structure comprising antiparallel beta-barrels in both S and P-domains. This model postulates a T = 3 dimer clustered capsid and is superficially compatible with our cryo-EM density map. We repeated this analysis (using the Phyre2 server^32^) and overlaid the resulting coordinates onto our reconstructed density map in UCSF Chimera^33^. This revealed that the shapes and orientations of the predicted P-domain structures matched poorly the computed density map (Fig. 4). Rigid body fitting of the predicted capsid protein coordinates was not successful. When S and P domains were docked independently however we achieved reasonable correlation in the S-domains, suggesting that the predicted eight-stranded antiparallel beta-barrel topology is most likely correct. Fitting of the P-domain at the CC position also achieved a reasonable result, although the domain was rotated and raised significantly from the S-domain. The dimeric P capsomere is also predicted to be broader than that which we see in our reconstruction. We were unable to achieve a satisfactory fit of the P domain at the AB position, owing to clashes of the docked coordinates.

Attempts to use a similar approach with SWISS-MODEL^34^ identified capsid proteins of other nodaviruses (Orsay virus^23^) or tombusviruses (carnation mottle virus^35^) as suitable templates for modelling (Supplemental Fig. 2). While these modelling experiments strongly point to a likelihood of the *Mr*NV capsid protein S-domain adopting an eight-stranded antiparallel beta-barrel topology, they have not allowed us to propose a model that is consistent with our intermediate resolution cryo-EM data.

In summary, our analysis of the structure of the *Mr*NV capsid has demonstrated that this virus is highly diverged from the alpha- and beta-nodaviruses, as the capsid shows a unique morphology characterized by the presence of thin blade-like dimeric P-domains, comprising the C-terminus of the capsid protein. These capsomeres come together to form a large linear super-structure on the capsid surface lying across the icosahedral two-fold symmetry axes. This feature is likely critical for virus attachment and entry. These data strongly support the assertion that *Mr*NV may belong to a new genus of the virus family *Nodaviridae*. Further study, to probe the structure of the *Mr*NV capsid protein at close to atomic resolution, could inform the development of peptide inhibitors to block the virus-host cell interaction. High resolution structural characterisation of the P-domain may also lead to the definition of candidate vaccines capable of eliciting a protective response in freshwater prawn.

## Methods

### Preparation of baculovirus stock

P1 stock of baculovirus was prepared according to the manufacturer’s protocol (Bac-to-Bac Baculovirus Expression System, Invitrogen) with some modifications. Briefly, the P1 stock was prepared by transfecting the *Spodoptera frugiperda* (*Sf*9) cells at a cell density of 8 × 10^5^ cells/well on a 6-well plate with Bacmid containing the *Mr*NV capsid protein gene and a 6× His-tag coding sequence. Following incubation at 27 °C for 72 hours, the medium containing the P1 baculovirus was collected. The remaining insect cells and large debris in the medium were removed by centrifugation at 500 × g for 5 min at 4 °C. The P1 stock of baculovirus was stored at 4 °C, protected from light. To prepare the P2 stock of baculovirus, *Sf*9 cells at a cell density of 2 × 10^6^ cells/well in a 6-well plate were infected with the P1 stock. The infected cells were incubated at 27 °C for 48 hours before the medium containing the P2 baculovirus stock was collected. The remaining insect cells and large debris in the medium were removed by centrifugation at 500 × g for 5 min at 4 °C. The P2 stock of baculovirus was stored at 4 °C before used.

### Expression and purification of His-tagged *Mr*NV capsid protein

*Sf*9 cells were grown at 27 °C in Sf-900 III SFM (Life Technologies, USA) supplemented with 4% (v/v) foetal bovine serum (FBS). When the cell density reached 1–2 × 10^6^ cells/ml, the cells were infected by 10% (v/v) of the recombinant baculovirus stock harbouring the *Mr*NV capsid protein gene and an N-terminal 6 × His-tag coding sequence (The His-tag was not however used as it was found not to be accessible). The culture was incubated for 4 days before the infected cells and the culture medium were separated by centrifugation at 500 × g for 5 min. The proteins in the culture medium were precipitated at 50% (w/v) ammonium sulphate saturation for 2 hours and the precipitated proteins were pelleted down at 12,000 × g for 20 min and resuspended in sodium phosphate buffer (77.4 mM Na_2_HPO_4_, 22.6 mM NaH_2_PO_4_; pH 7.4). The proteins were then separated by sucrose gradient ultracentrifugation [10–40% (w/v)] at 150,000 × g for 4.5 hours at 4 °C with a SW 40 Ti rotor (Beckman Coulter, USA). This was followed by fractionation into 1 ml fractions. The fractions which contained the *Mr*NV capsid protein, as analysed by sodium dodecyl sulphate-polyacrylamide gel electrophoresis (SDS-PAGE), were dialysed in sodium phosphate buffer as above. The purified protein was concentrated by centrifugation using a centrifugal concentrator with molecular cut-off 50 kDa (Microsep centrifugal devices, Pall, USA) at 4000 × g at 4 °C. The final protein concentration was determined by the Bradford assay.

### Cryo-electron microscopy

Purified *Mr*NV capsid protein at approximately 1 mg/ml was prepared for cryogenic transmission electron microscopy using an FEI Vitrobot Mk IV. Four μl of VLP preparation was applied to freshly glow-discharged quantifoil holey carbon support films (R2/2; Quantifoil, Jena, Germany), blotted for 4 seconds and plunged into liquid ethane^36^. Vitrified samples were viewed at low-temperature (around 98 K) and under low electron dose conditions using a JEOL 2200 FS cryo-microscope operated at 200 kV. Samples were held in a Gatan 626 cryo-stage. Micrographs were recorded at 50,000 × magnification on a Direct-Electron DE20 DDD camera giving a sample rate of 1.09 Å/pixel. Three-second exposures, at an electron dose rate of ~20 e/Å^2^/s, were captured in movie mode running at 20 frames per second.

### Computational image reconstruction

Micrograph movies containing 60 frames were processed to calculate a 3D reconstruction using Relion-2.0^37^. Initially movies were corrected for specimen movement using MotionCorr^38^ and defocus was estimated using GCTF^39^. An initial set of ~1000 particles was manually picked and processed to calculate a 2D average suitable for automated particle picking. Following automated particle picking 2D classification was used to select intact particles for 3D reconstruction. Representative 2D class-averages are shown in Supplemental Fig. 3. For 3D classification, a starting model was prepared from the X-ray crystallographic coordinates of the Flock House virus capsid protein (PDB 4FTB – DOI 10.2210/pdb4ftb/pdb) using the EMAN program pdb2mrc^40^. In line with recommended procedures the starting model was low-pass filtered to 60 Å to limit the effects of model-bias. A subset of particles was selected from the 3D class that yielded the highest resolution and further refined. Following refinement, per-particle movie correction was performed using only frames 2–20, to limit the impact of radiation damage. Finally, a post-processing b-factor of −890 Å^2^ was estimated and applied to the reconstruction using Relion^41^. To satisfy ourselves that our model was not affected by model-bias, we also performed a 3D autorefine analysis of our final dataset using a Gaussian sphere as a starting model, yielding essentially identical results (Supplemental Fig. 4).

The calculated 3D reconstruction was visualized in UCSF chimera and IMOD^33, 42^. The map has been deposited in the electron microscopy data bank, with accession number EMD-3655.

## Electronic supplementary material

Supplemental figures 1 to 4
