# Supplementary material for: Cryo-Electron Microscopy Structure of the Macrobrachium rosenbergii Nodavirus Capsid at 7 Angstroms Resolution
Source: Sci Rep. 2017 May 18;7:2083. doi: 10.1038/s41598-017-02292-0 (PMC5437026; doi:10.1038/s41598-017-02292-0)
Supplement: Supplementary file 1 — Supplemental figures 1 to 4 [file 41598_2017_2292_MOESM1_ESM.pdf]

Cryo-Electron Microscopy Structure of the *Macrobrachium rosenbergii* Nodavirus Capsid at 7 Angstroms Resolution

Kok Lian Ho, Chare Li Kueh, Poay Ling Beh, Wen Siang Tan, David Bhella

Supplemental figures

Figure S1.

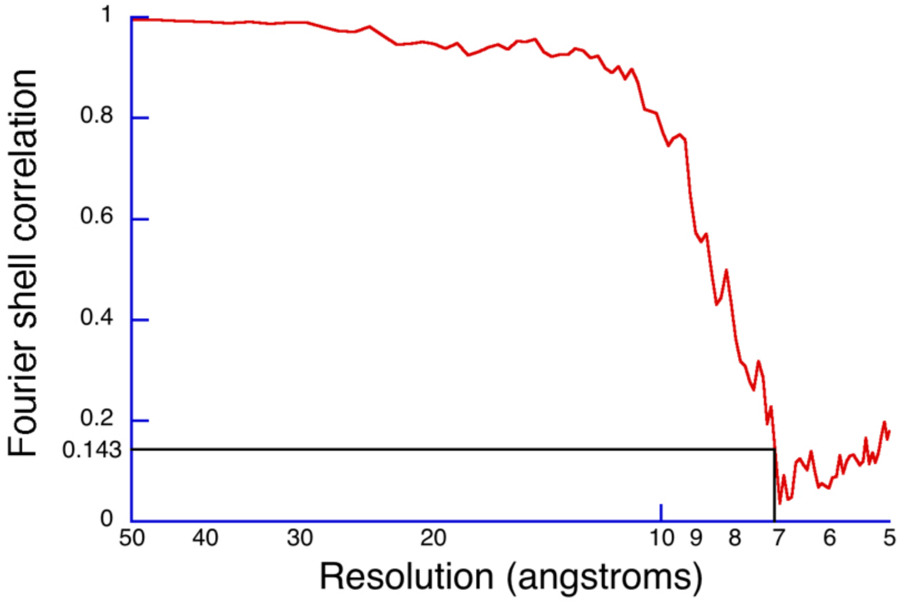

Gold-standard Fourier shell correlation plot for the *Mr*NV 3D reconstruction, indicating a resolution of 7 angstroms was achieved assuming an FSC cut-off of 0.143.

Figure S2

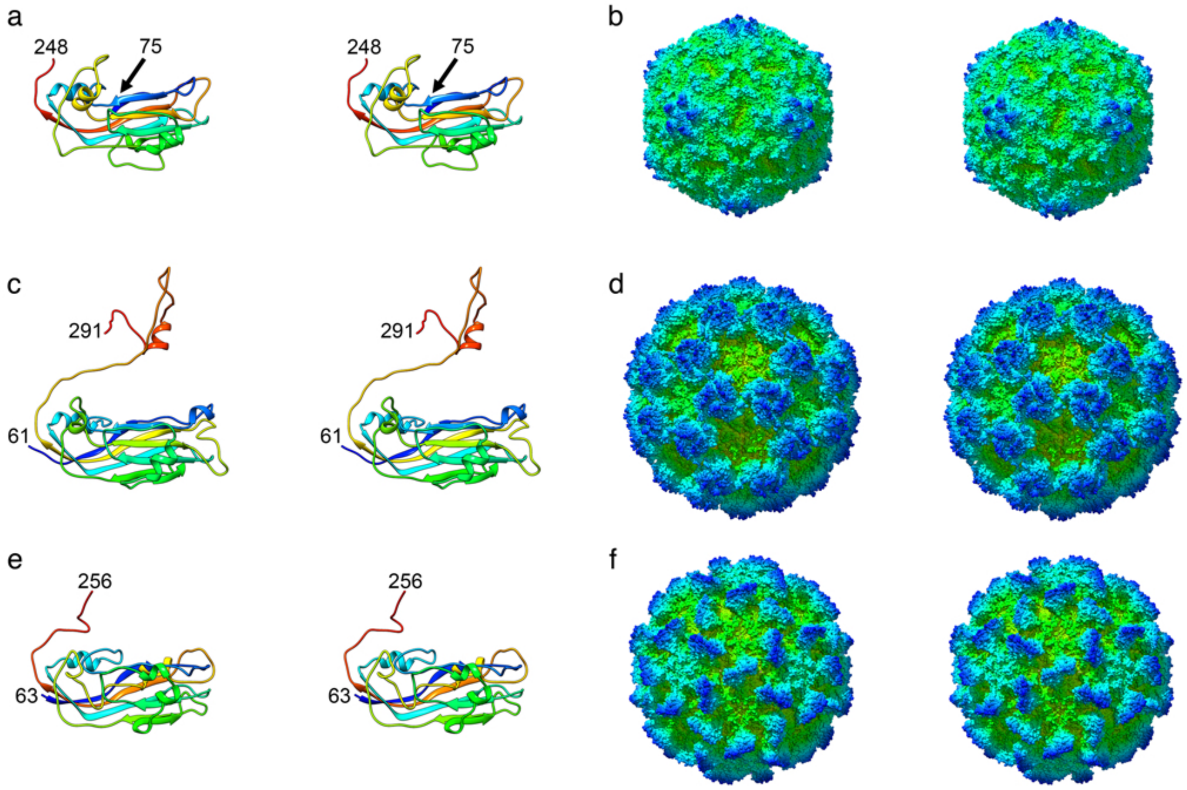

Structure prediction was performed using the known structures of several plant and animal viruses from both *nodaviridae* and *tombusviridae* determined by X-ray crystallography. The previously published homology modelling against cucumber necrosis virus – PDB ID 4LLF is presented in the article as figure 4. Here three other viruses were used for modelling. Left-hand panels show ribbon diagrams of the homology models, while the right-hand panels show solvent excluding surface representations of the viral capsid structures that informed the homology model. (a,b) cocksfoot mottle virus 2 – PDB ID 1NG0, (c,d) Orsay virus – PDB ID 4NWV and (e,f) carnation mottle virus PDB ID 1OPO. These models show a common eight-stranded anti-parallel beta-barrel topology in the S-domain. The N and C terminal residues for each ribbon diagram are labelled, indicating the extent of the capsid sequence that was modelled.

Figure S3

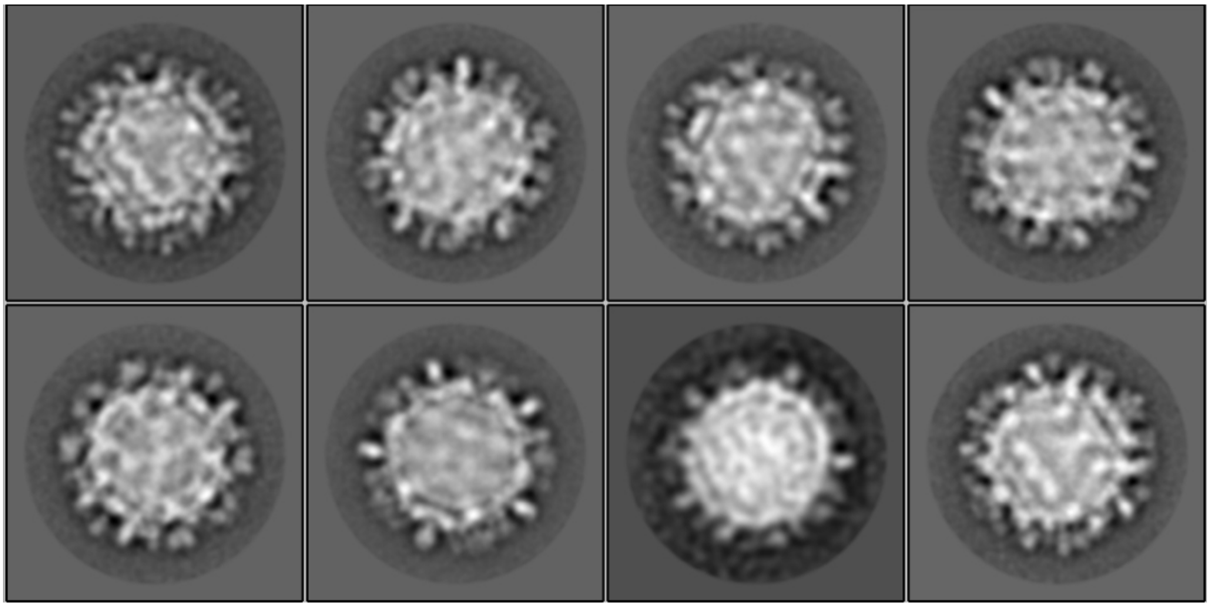

A subset of class-averages of MrNV VLPs showing the presence of pronounced spikes on the capsid surface.

Figure S4a

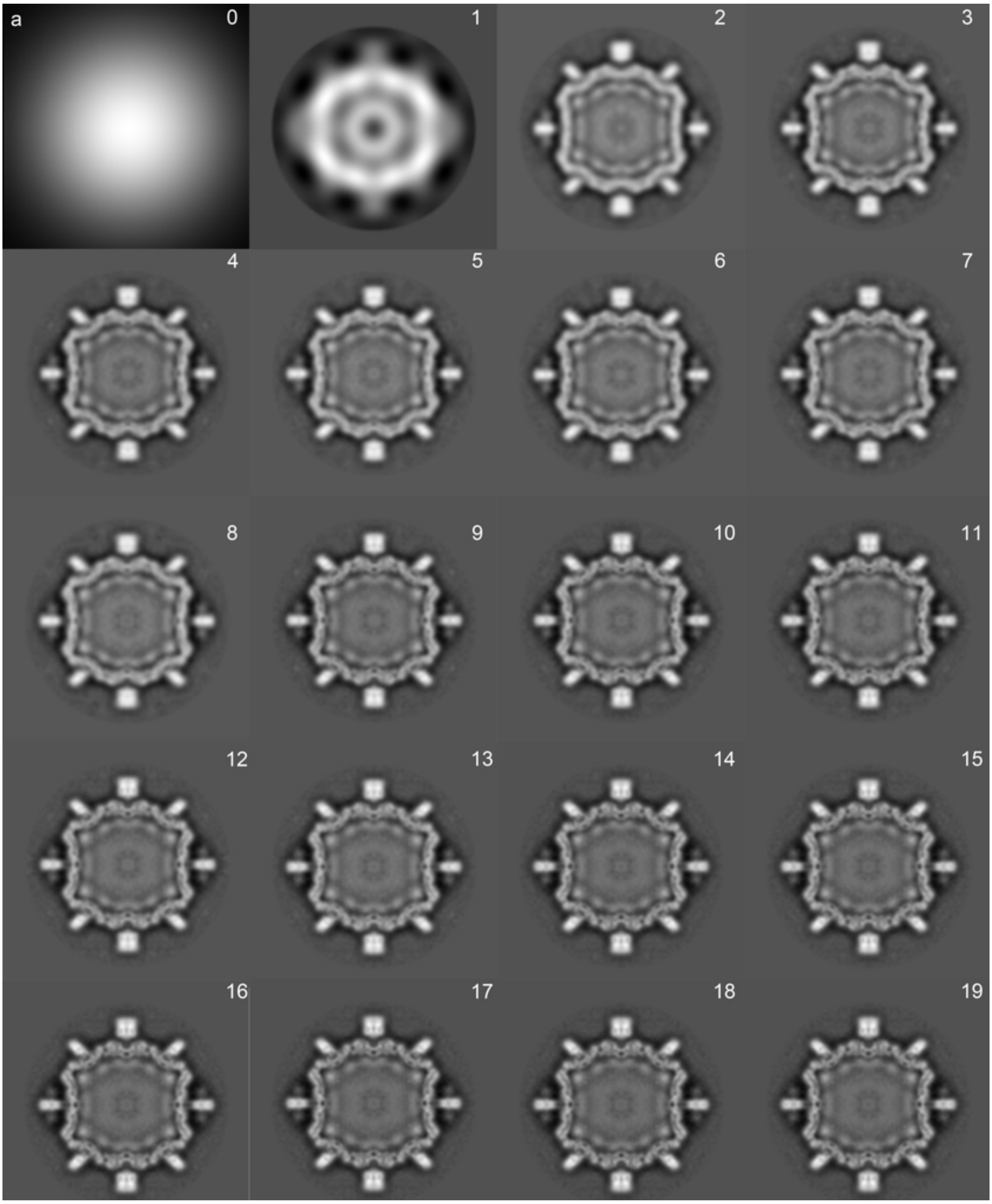

Figure S4b

b

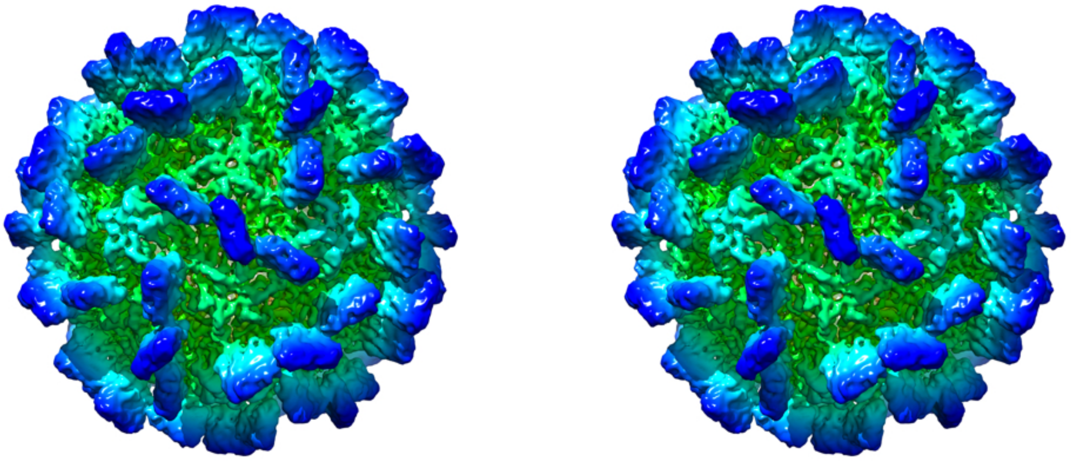

To ensure that our three-dimensional reconstruction was not the product of a model-biased refinement, we prepared a reference-free reconstruction. A subset of binned particles was selected following 2D and 3D classification in Relion. These were then subjected to a 3D auto-refine procedure for which a Gaussian sphere was used as a starting model. (a) Central sections through reconstructions calculated from half-datasets at each iteration of the refinement are given, iteration 0 represents the input starting model. (b) A surface view of the 7-angstrom resolution reconstruction that was determined by iteration 19.
